# Galaxy Portal: interacting with the galaxy platform through mobile devices

**DOI:** 10.1093/bioinformatics/btw042

**Published:** 2016-01-27

**Authors:** Claus Børnich, Ivar Grytten, Eivind Hovig, Jonas Paulsen, Martin Čech, Geir Kjetil Sandve

**Affiliations:** 1 Biomedicial Informatics, Department of Informatics, University of Oslo, Oslo, Norway,; 2 Department of Tumor Biology, Institute of Cancer Research, Oslo, Norway,; 3 Department of Cancer Genetics and Informatics, Radium Hospital, Part of Oslo University Hospital, Oslo, Norway and; 4 Department of Biochemistry and Molecular Biology, Penn State University, University Park, Pennsylvania 16802, USA

## Abstract

**Summary**: We present Galaxy Portal app, an open source interface to the Galaxy system through smart phones and tablets. The Galaxy Portal provides convenient and efficient monitoring of job completion, as well as opportunities for inspection of results and execution history. In addition to being useful to the Galaxy community, we believe that the app also exemplifies a useful way of exploiting mobile interfaces for research/high-performance computing resources in general.

**Availability and implementation**: The source is freely available under a GPL license on GitHub, along with user documentation and pre-compiled binaries and instructions for several platforms: https://github.com/Tarostar/QMLGalaxyPortal. It is available for iOS version 7 (and newer) through the Apple App Store, and for Android through Google Play for version 4.1 (API 16) or newer.

**Contact**: geirksa@ifi.uio.no

## 1 Introduction

Present-day biomedical research often depends on compute-intensive data processing and analysis at different stages of an investigation. Galaxy (Goecks *et al.*, 2010) is a widely used platform for genome analysis, providing a web-based interface to initiate and monitor computations executed on a server. It can, in certain situations, be more convenient to access Galaxy through a mobile device than through a computer for quick inspection of analysis results or to check which underlying tools and parameter settings were used to produce a given result. Also, monitoring execution status may be more convenient through a mobile device. As biomedical analyses may involve several computational steps, each running for a long and *a priori* undetermined time period, it may be important to frequently check for job completion so as to know when subsequent steps should be initiated. Although the standard web interface of Galaxy can be used through a mobile device, it is not well suited for a small screen and touch-based interface.

**Fig. 1:**
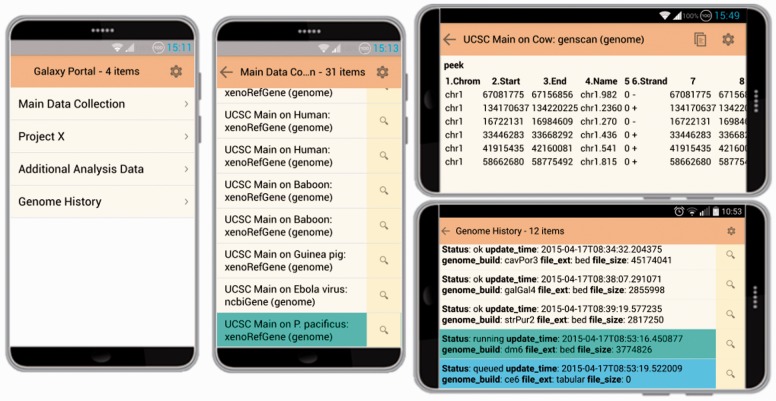
User interface on Android, showing left: histories, middle: jobs with a running item, and right: landscape oriented examples

Galaxy Portal is an open source interface to the Galaxy system through smart phones and tablets. Rather than supporting the full functionality of Galaxy, the Galaxy Portal app limits itself to monitoring of job completion, re-running of analyses and inspection of results. The purpose is to provide a simple and efficient interface that can be accessed from almost anywhere at any time through a mobile device. The app is responsive and low-bandwidth, with data presentation tailored to fit a small screen (Fig. 1).

## 2 Methods

The app was developed with the Qt cross-platform framework (http://www.qt.io), which compiles to native iOS and Android apps for optimal performance. The Qt framework is C ++  based, but the app was primarily developed by using the Qt Modelling Language (QML), with much of the logic of QML constructs coded in JavaScript. Qt was chosen because of the possibility of rapidly developing an app with a touch-based interface using QML and JavaScript, while retaining the performance and flexibility of a native app.

Access to Galaxy data is achieved by using the RESTful Galaxy API (Sloggett *et al.*, 2013) to retrieve JSON formatted data, which keeps bandwidth use low. Due to the use of the API, the app can connect to any Galaxy site, or any tool built on top of the Galaxy source, as long as the site is not behind additional access restriction (the current version of the app does not handle proxy access authentication). The app allows researchers to keep a list of connections to Galaxy instances, to enable easy switching between sites.

The app provides a touch-based interface that scales according to screen size and resolution. To reduce the security risk, the password is not stored and a passcode can be created to prevent unauthorized access through the app.

## 3 Results

The Galaxy Portal has been tailored to provide responsive monitoring, results inspection and parameter modification of Galaxy jobs as follows:

***Responsive navigation of histories and elements***: the app exploits the limited screen size by providing large elements for browsing histories and elements within a selected history.

***Detailed (and customizable) view of job metadata, including result preview***: tapping a history element cycles through a configurable selection of metadata for the element.

***Viewing content of history elements***: datasets and results in plain text or html can be viewed through the app.

***Re-running of jobs***: from a history element it is possible to see which tool was used to generate the element and the parameter selections that were made. It is also possible to re-run the tool, either with the same or with modified selections.

***Access to shared datasets***: the app provides access to histories shared by other users.

***Colo****u****r-coded status indicators***: the app visually distinguishes between queued, running, finished and failed runs through the use of different coloured history elements.

***Audible notifications***: the user can optionally receive an audio notification when the status of a job changes (also works when app is running in background).

***Configurable status polling***: the app can be set to refresh job status (by polling the server) at fixed intervals (of customizable frequency). This also works when app is running in background.

***Storing user credentials (except password)***: the app allows storing the URL, username and API key used to access a Galaxy instance.

***Managing multiple Galaxy instances***: the app allows storing credentials for multiple Galaxy instances, and easy switching between these stored instances.

## 4 Discussion

The standard Galaxy web interface relies on small interactive elements spread across the screen, and is not well suited for the small screens and touch interfaces of mobile devices. The Galaxy Portal app therefore employs a radically different user interface, where not only the layout and graphical elements are different, but rather the whole way of interacting. For instance, history elements are browsed by employing the full screen for selecting a history and an element in succession, while the standard web interface lists history elements in a small part of the screen and relies on small buttons (‘Options’) to switch between histories. Such a distinct behaviour has been achieved by defining a separate GUI for the app that communicates with the Galaxy instance through its RESTful API.

Not all of Galaxy’s functionality is accessible through the app in the current version. As the Galaxy system has a large developer community, we expect and welcome contributions, which can for instance be made as pull requests to the GitHub-hosted open source codebase of the app. A natural future extension would be the possibility to launch new jobs through the app. Functionality for handling external access authentication would allow access to Galaxy instances that are for instance behind proxy authentication schemes. The functionality for inspecting results and re-running jobs is in the current version limited to a core subset of formats (currently plain text and html) and user interface elements, and it would be interesting to add support for viewing additional formats (e.g. dataset visualizations) and for re-running tools that utilize more complex user interfaces.

Galaxy Portal is distributed through centralized app marketplaces as a native app. An alternative would be to provide corresponding functionality through a browser-based interface on a mobile device. While it is beyond the scope of this article to discuss apps versus web pages, we do note that native apps have gained broad popularity in recent years. Having a native app makes Galaxy Portal more visible, responsive and easy to use, whereas a pure web solution would rely on users to go to or bookmark a website. Also, a native app allows access to device-specific features such as audible notifications, including notifications when the app is running in the background.

At the same time, the dependence on distribution channels such as Apple App Store and Google Play is debatable, as it represents an additional challenge for software longevity (although the source code of Galaxy Portal itself is freely available through GitHub). We note that wrapping a web app in a native container (through, e.g. the PhoneGap framework) enables visibility of an app, but has the same dependence of distribution channels as a native app. We also note that Section 2.12 in the current app review guidelines state that web sites bundled as apps may be rejected, bringing into question how reliable such an approach might be. For Galaxy Portal, we consider the advantages of having a native app to outweigh the disadvantages related to reliance on distribution channels. We have consequently allocated funds to pay for Apple App Store distribution through the coming 5 years (this is the only distribution channel requiring a small periodic fee to continue distribution of apps), and consider this to be a satisfactory solution.

Galaxy Portal has been developed with an intention of becoming a community project, and we encourage further development of the app through the open source repository at GitHub.

## Funding

Efforts of the Galaxy Team and collaborators were instrumental for making this work happen. Martin Čech and the Galaxy Team was supported through grant number HG005542 from the National Human Genome Research Institute, National Institutes of Health as well as grants HG005133, HG004909 and HG006620 and NSF grant DBI 0543285. Additional funding is provided by Huck Institutes for the Life Sciences at Penn State and, in part, under a grant with the Pennsylvania Department of Health using Tobacco Settlement Funds.

*Conflict of Interest:* none declared.
